# Supplementary material for: Longitudinal tracking of acute kidney injury reveals injury propagation along the nephron
Source: Nat Commun. 2023 Jul 21;14:4407. doi: 10.1038/s41467-023-40037-y (PMC10362041; doi:10.1038/s41467-023-40037-y)
Supplement: Supplementary file 1 — Supplementary Information [file 41467_2023_40037_MOESM1_ESM.pdf]

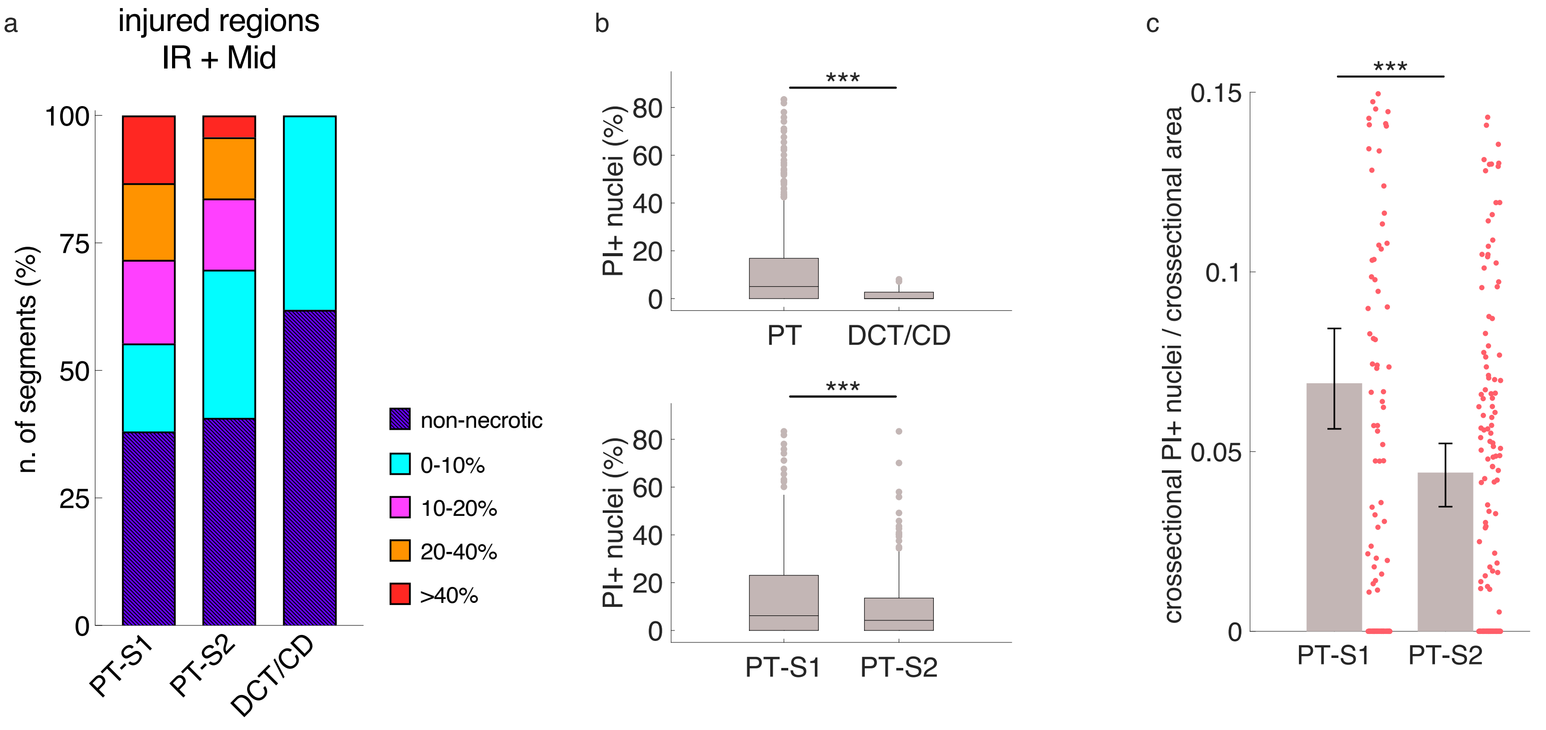

Supplementary Figure 1: Necrotic injury distribution along the nephron in IR and Mid areas. Displayed data derived from tubule segments from IR and Mid regions of partial IRI kidneys. (a): Necrotic injury distribution across partial IRI regions clustered by percentage of segments with indicated threshold of propidium iodide (PI)+ nuclei (% of total nuclei/ segment). (b): Volumetric quantification of PI+ nuclei (% of total nuclei/ segment). (n = 484, 42, 226, 258 segments from 7 mice for PT, DCT/CD, PT-S1, and PT-S2, respectively); boxplot, line at median, edges at 25th and 75th percentiles, whiskers from min to max, individual points considered outliers. Statistical test: linear mixed-effect model, p values from two-sided test (extended statistics.t). (c): Quantification of PI+ nuclei determined from a 2D plane at the highest cross-sectional area within each tubular segment, normalized by respective cross-sectional area achieves similar results as when quantifying total number of PI+ cells in 3D (n = 164, 176 PT-S1 and PT-S2 segments from 6 mice, respectively); mean ± 95% CI with scatterplot. Statistical test: linear mixed-effect model, p values from two-sided test (extended statistics.t). *: $p < .05$; **: $p < .01$; ***, $p < .001$.

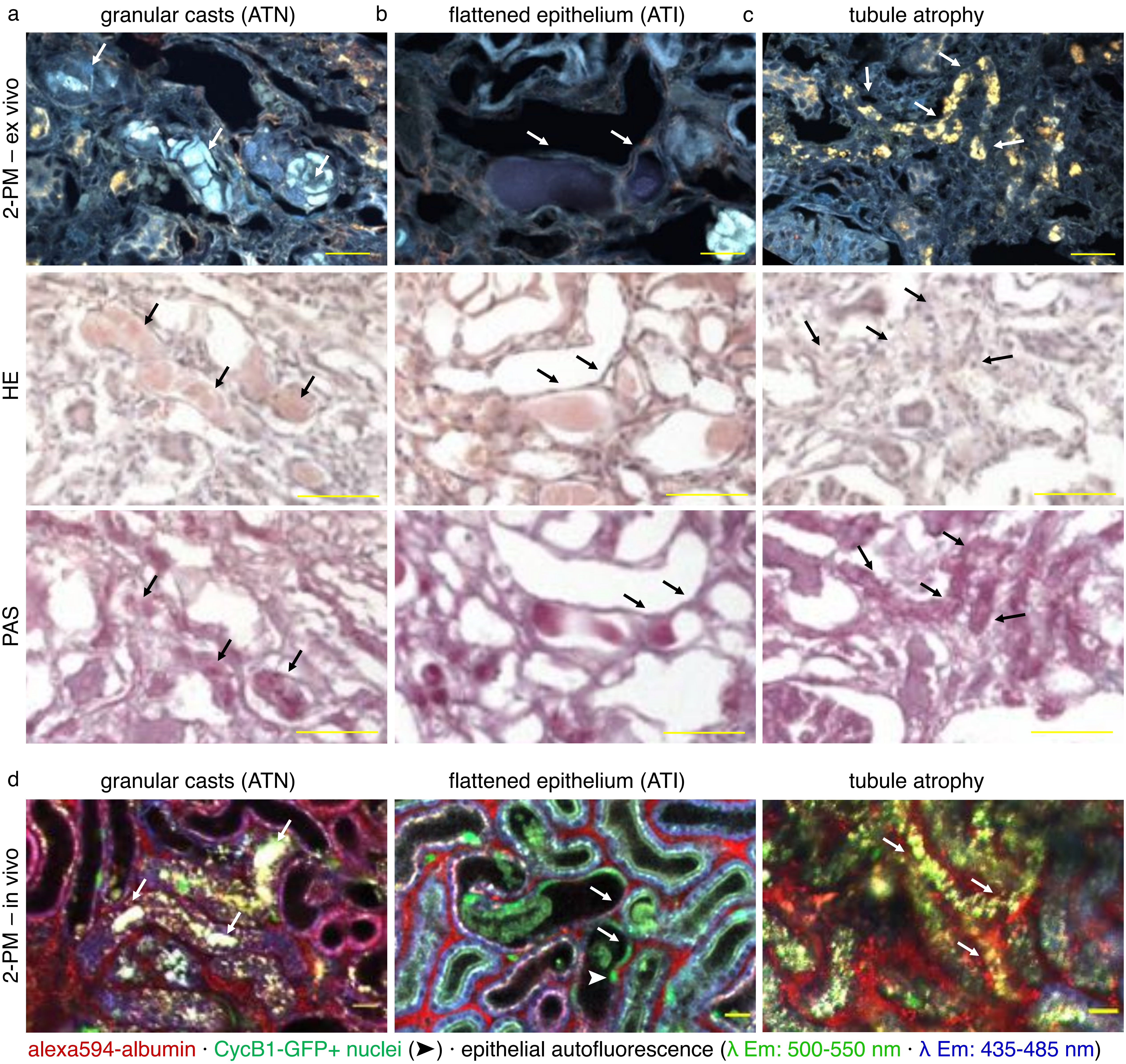

Supplementary Figure 2: Correlation of tubular remodeling processes visualized via 2-photon microscopy (2PM) and via HE/PAS staining. (a-c): Representative correlative microscopy of serially cut 6 µm thick frozen sections (n = 3 partial IRI kidneys), which were imaged unstained via 2PM and via light microscopy after HE and PAS staining, respectively. Arrows point out the same tubules in 2PM and light microscopy images, displaying either granular casts, indicative of acute tubule necrosis (ATN) (a), flattened and simplified tubule epithelium, indicative of acute tubule injury (ATI) (b), and tubule atrophy (c), respectively. Scale bars: 25 µm. (d): Representative in vivo 2PM images of granular casts, flattened epithelium, and tubule atrophy. Scale bars: 25 µm.

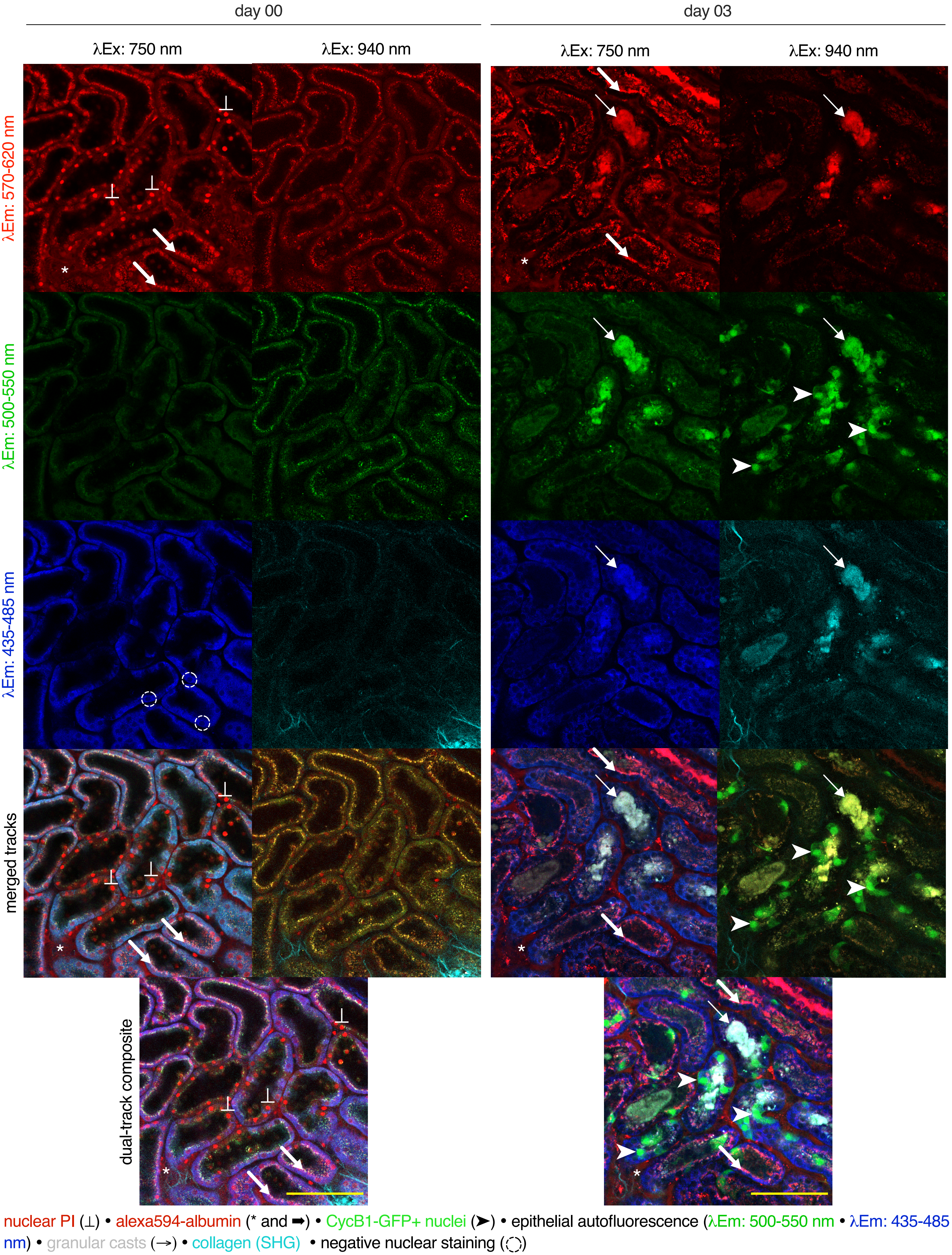
day 00
day 03
λEx: 750 nm
λEx: 940 nm
λEx: 750 nm
λEx: 940 nm
λEm: 570-620 nm
λEm: 500-550 nm
λEm: 435-485 nm
merged tracks
dual-track composite
nuclear PI (⊥) • alexa594-albumin (* and ➡) • CycB1-GFP+ nuclei (➤) • epithelial autofluorescence (λEm: 500-550 nm • λEm: 435-485 nm) • granular casts (→) • collagen (SHG) • negative nuclear staining (◌)

Supplementary Figure 3: Dual track 2-photon intravital imaging of tubule injury and remodeling in partial IRI kidneys. Emission light was collected over 3 separate detection channels using a dual-track excitation protocol (λ_{Ex} = 750 and 940 nm). Exemplary images of individual detection channels and merged tracks for day 00 and day 03 after partial IRI are shown. Dual-track composites have been used to display images throughout the manuscript. Merged 750 nm track channel data was used to assess total number of nuclei and number of PI+ nuclei (% of total nuclei/ segment) in individual segments in 3D, as well as albumin reuptake capacity. At λ_{Ex} = 750 nm, tubule epithelia were identified by strong blue and green epithelial autofluorescence which excluded nuclei and allowed counting of total nuclei number based on negative staining (day 0, white circles). PI+ nuclei (day 0, ⊥) counting was based on strong red nuclear fluorescence emitted at λ_{Ex} =750 nm. In addition, weaker red alexa594-albumin fluorescence identified peritubular vessels in the renal interstitium (asterisk) and albumin reuptake in the apical membrane of the proximal tubules (day 3, bold arrows). Granular casts (day 03, arrows) appeared as unspecific luminal signal across all tracks and channels. Merged 940 nm track channel data was used to assess number of GFP+ nuclei (% of total nuclei/ segment) (arrowheads) in individual segments in 3D. At λ_{Ex} = 940 nm, GFP emitted a strong nuclear (in part cytosolic) specifically green signal (arrowheads) that could be clearly differentiated from weak green tubule epithelial autofluorescence. Scale bar: 100 µm.

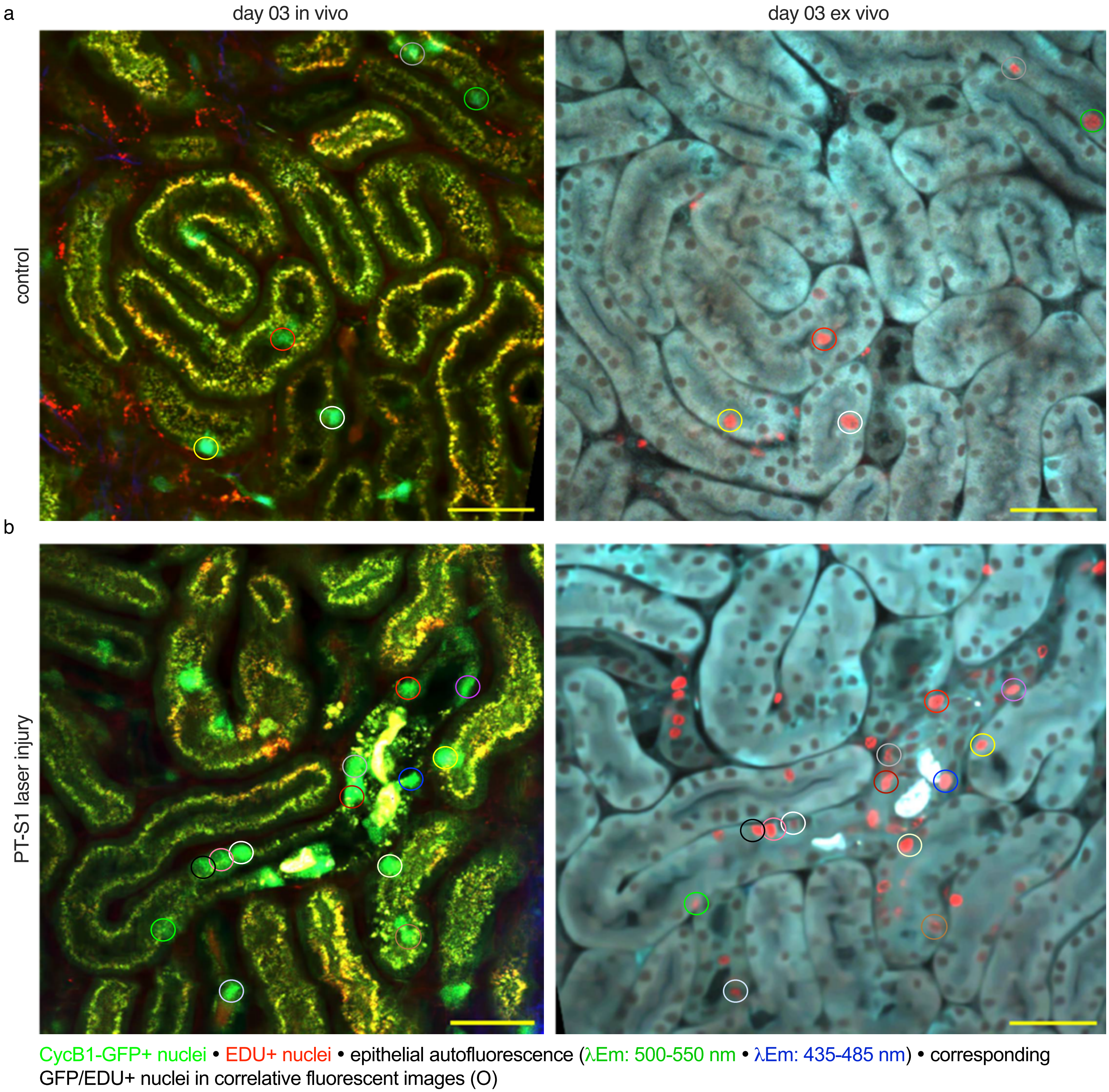

Supplementary Figure 4: EDU incorporation validates CycB1-GFP indicated cell proliferation. Correlative microscopy of in vivo GFP-expression as visualized via intravital 2-photon microscopy and nuclear EdU incorporation in proliferating cells as visualized ex vivo through Alexa647 conjugation and imaged using confocal microscopy. Representative correlative images display cortical kidney regions of CycB1-GFP kidney control regions (a) and regions 3 days after laser-induced selective PT-S1 injury (b). Corresponding GFP-expressing and EdU-incorporating cells are marked with color-coded rings in correlated microscopy images. Scale bar: 50 µm.

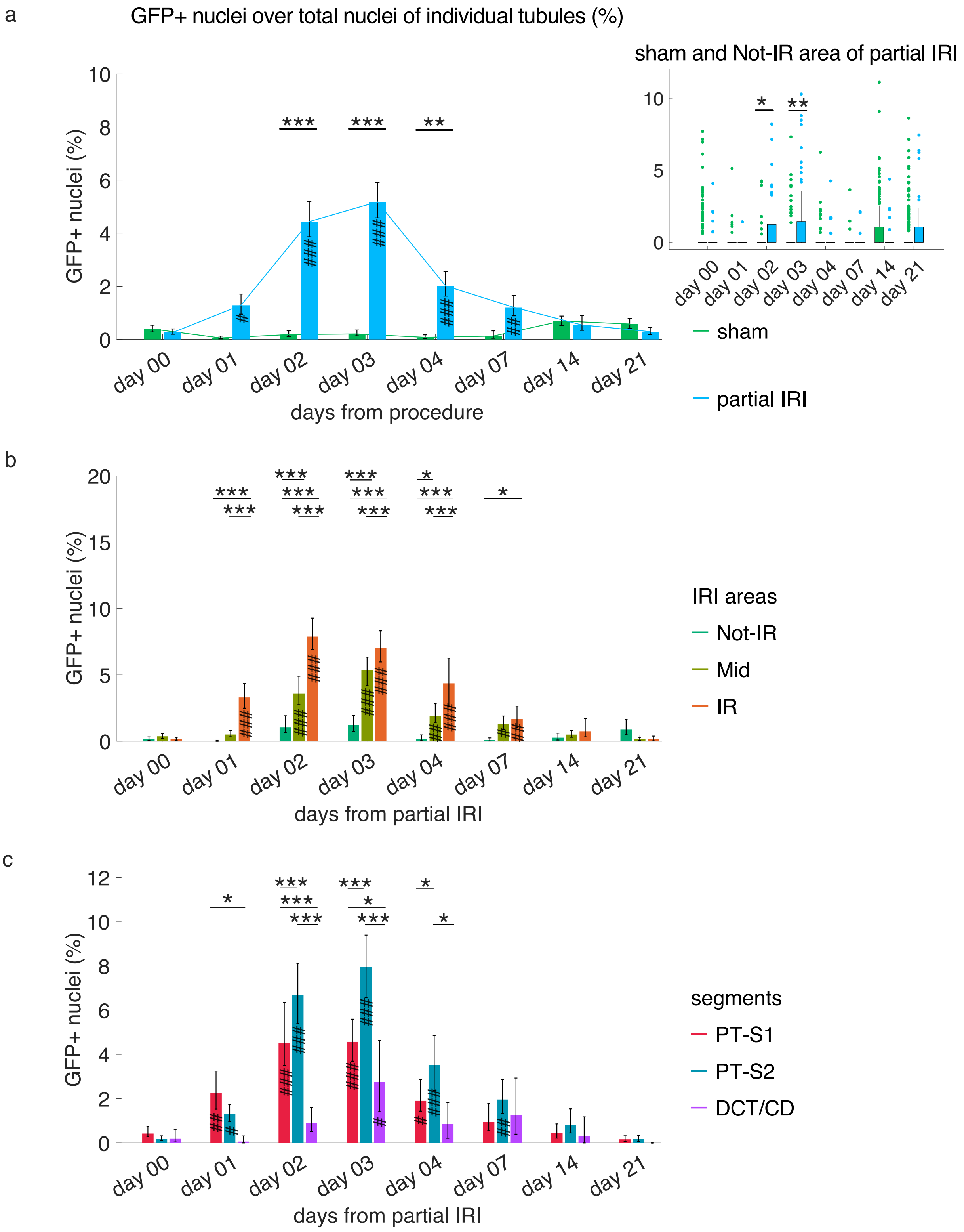

Supplementary Figure 5: Dynamic epithelial proliferation in partial IRI kidneys. Dynamic volumetric quantification of GFP-expression (% of total nuclei/ segment) across different experimental groups (a**:** sham and partial IRI), different injury regions of partial IRI kidneys (b: Not-IR, Mid, and IR), and different tubule segments (c: PT-S1, PT-S2 and DCT/ CD). Same data set as in fig. 3b-d from n = 3 sham and 9 partial IRI mice; mean ± 95% CI with scatterplot. Statistical test: linear mixed-effect model, p values from two-sided tests (extended statistics.j). *: significant difference between groups on the same day. #: significant difference within group when compared to respective day 0. */#: $p < .05$; **/##: $p < .01$; ***/ ###, $p < .001$.

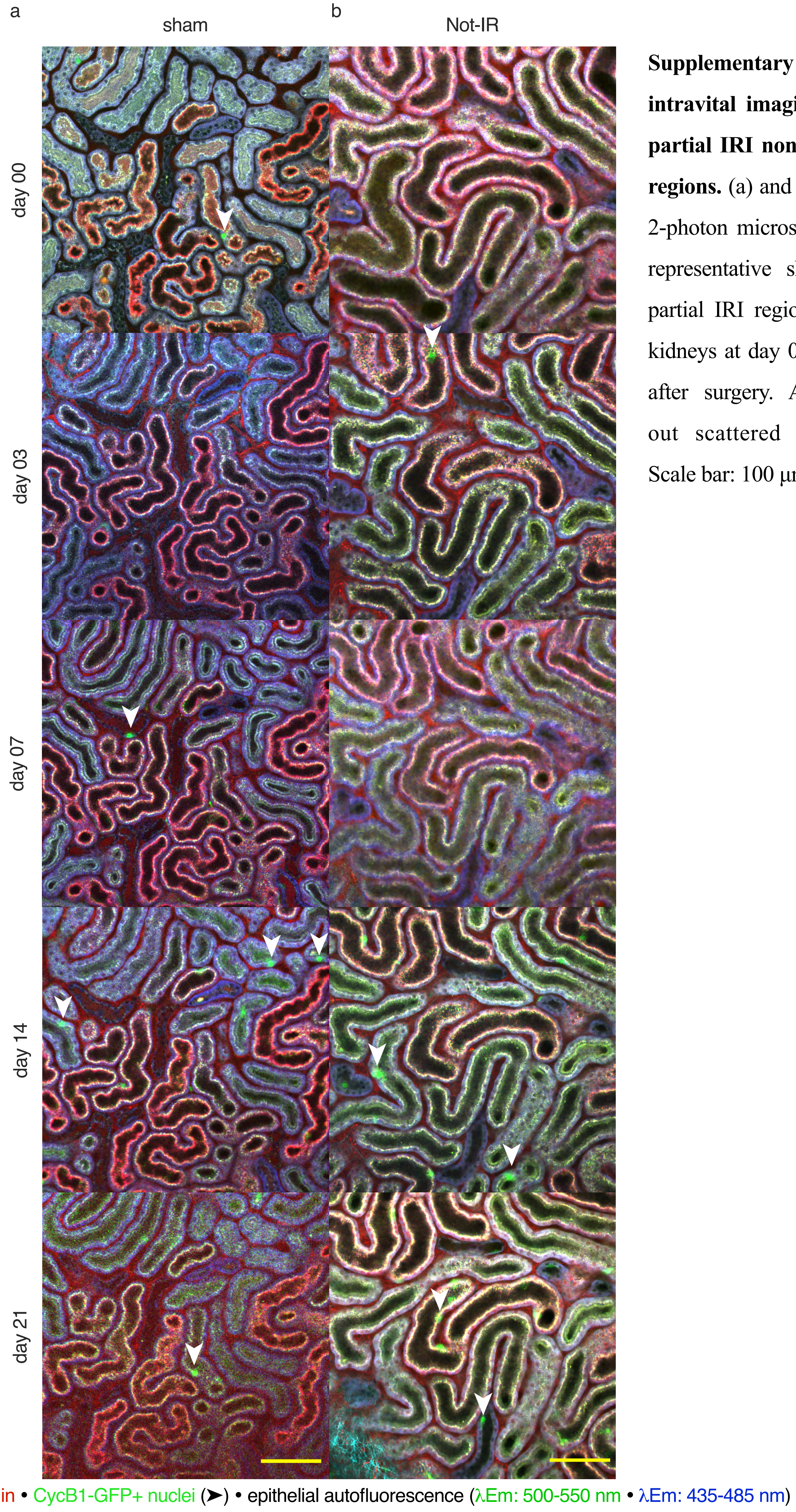

Supplementary Figure 6: Serial intravital imaging of sham and partial IRI non-ischemic kidney regions. (a) and (b): Serial in vivo 2-photon microscopy images of a representative sham and Not-IR partial IRI region in CycB1-GFP kidneys at day 0, 3, 7, 14, and 21 after surgery. Arrowheads point out scattered GFP-expression. Scale bar: 100 μm.

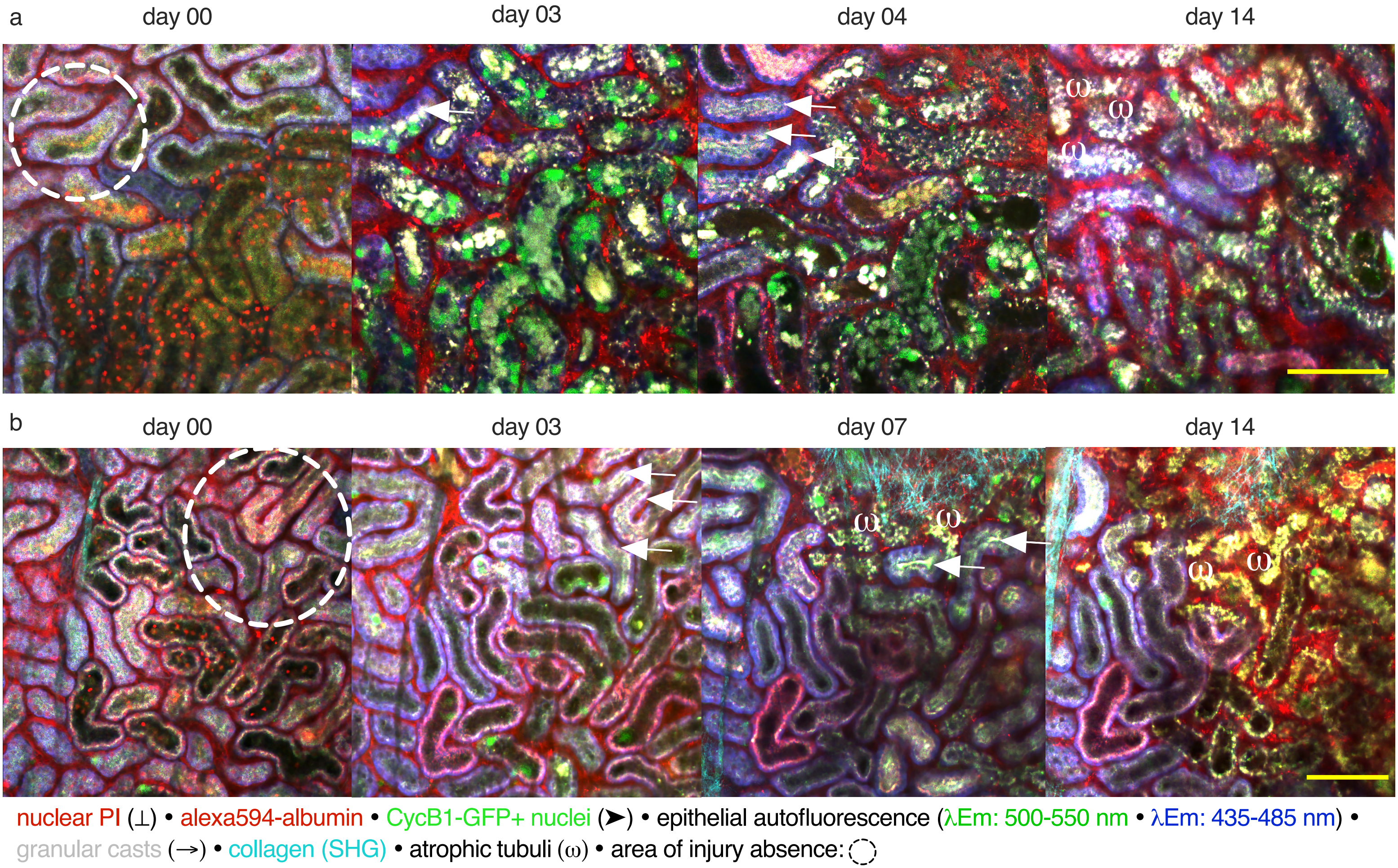

Supplementary Figure 7: Tubular morphological changes associated with tubule atrophy in non-necrotic segments. (a) and (b): Representative serial in vivo 2-photon microscopy images of ischemic regions (IR) in partial IRI CycB1-GFP kidneys over 2 weeks. Scale bar: 100 µm. Encircled regions mark populations of initially non-necrotic tubule segments (PI-negative at day 0) in close vicinity of necrotic tubule segments, which later accumulate granular casts (arrows) and eventually turn atrophic (ω).

a

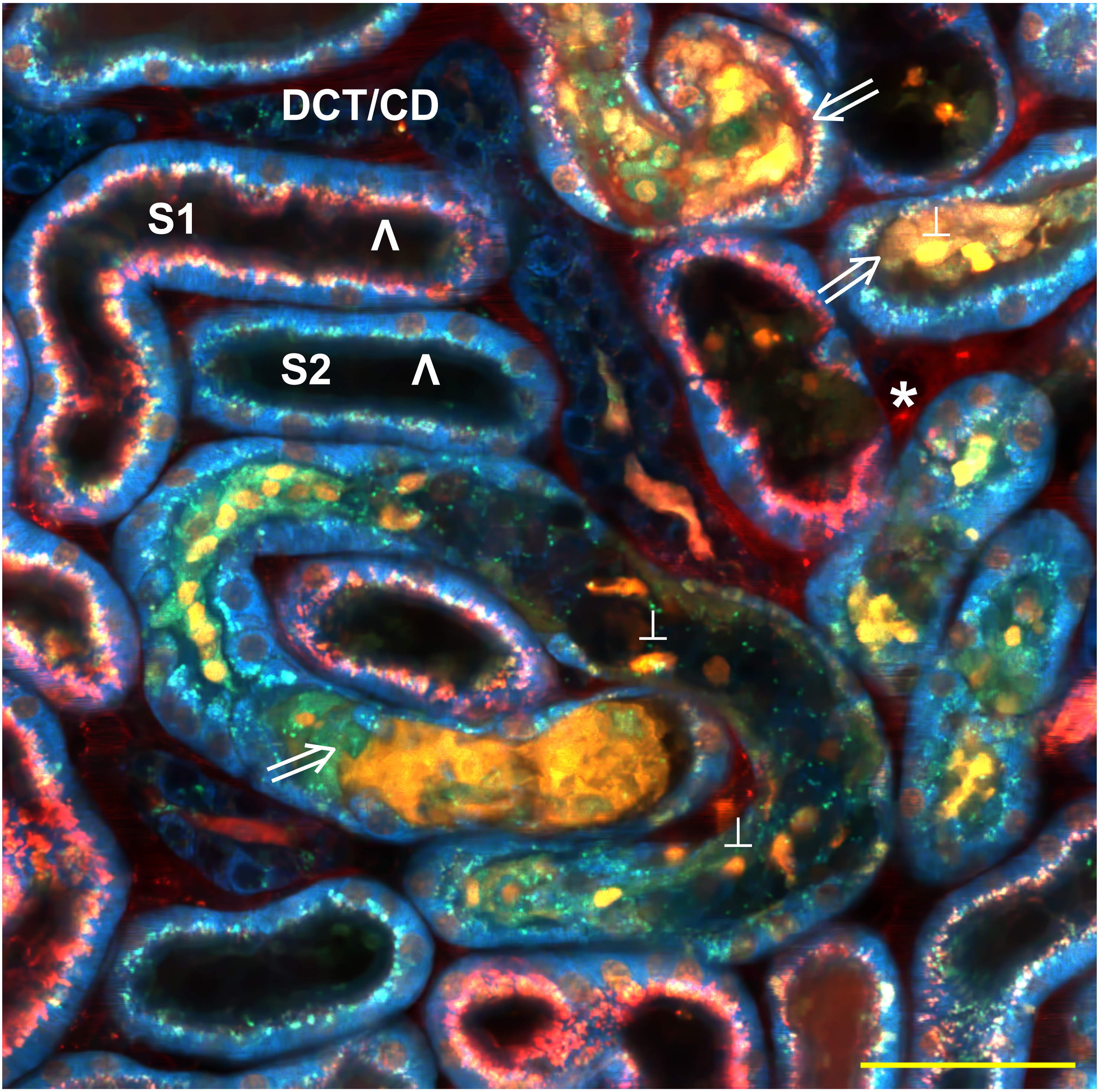

Intact apical membrane (∧) • necrosis (⇒) • nuclear PI (⊥) • alexa594-albumin • beginning edema (*) • epithelial autofluorescence (λEm: 500-550 nm • λEm: 435-485 nm). Scale bar: 50μm

Supplementary Figure 8: High-resolution image of figure 1h. In vivo 2-photon image acquired 6 hours after partial IRI displays heavily necrotic tubule segments (⇒) with luminal accumulation of PI+ cells (⊥), which are surrounded by several structurally intact tubule segments (∧). Note absence of fibrotic tissue remodeling but beginning formation of edema (*) adjacent of necrotic tubule segments. S1: proximal S1 tubule segment. S2: proximal S2 segments. DCT/CD: distal convoluted/collecting duct tubules. Scale bar: 50 μm.

a

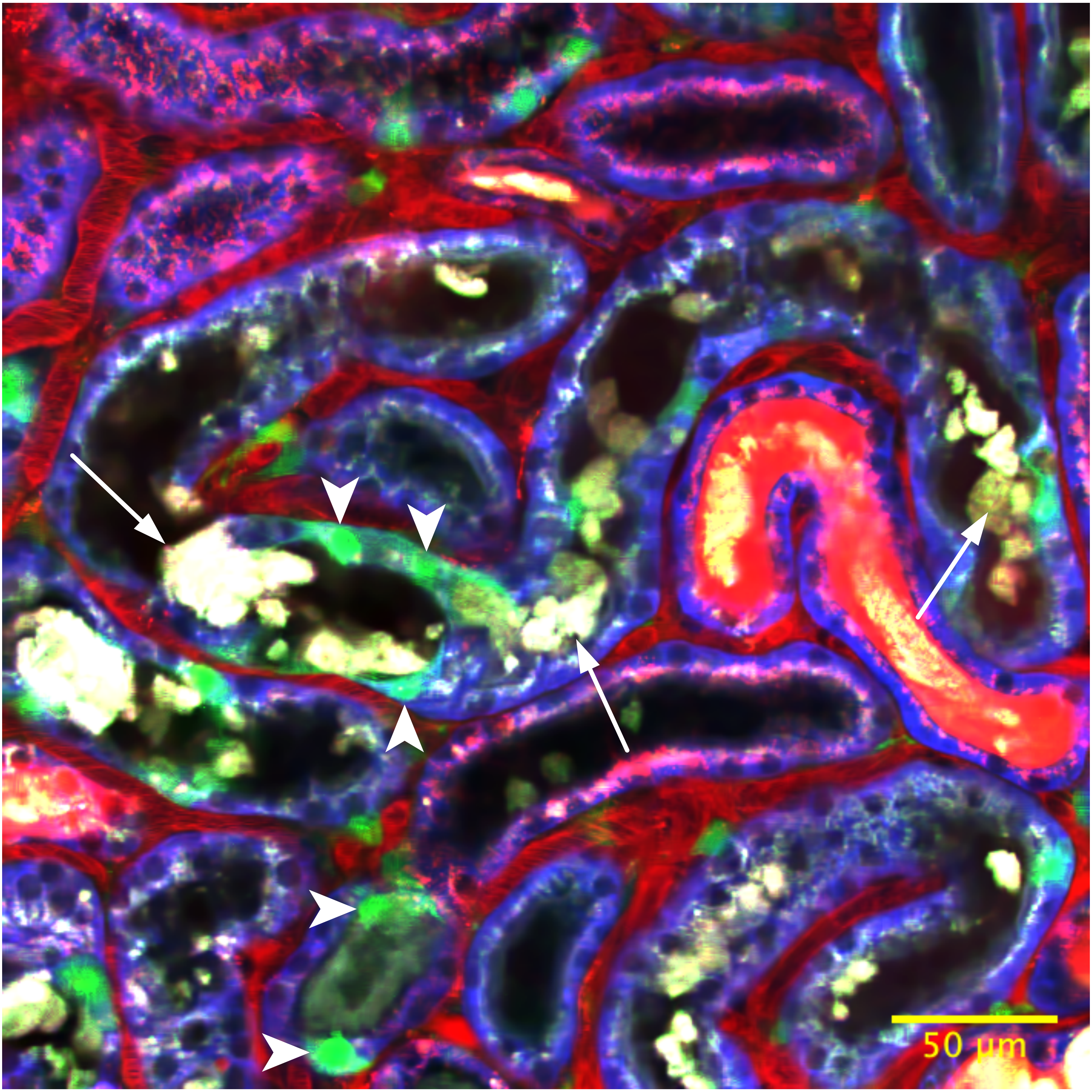

alexa594-albumin • CycB1-GFP+ nuclei (➤) • epithelial autofluorescence (λEm: 500-550 nm • λEm: 435-485 nm) • granular casts (→)

Supplementary Figure 9: High-resolution image of figure 4b. In vivo 2-photon microscopy image of a representative Mid region in a CycB1-GFP reporter kidney at day 2 after partial IRI. Scale bar: 50 µm.

Extended Statistical Analyses

Detailed information for the statistical tests used, references with letter to the specific statistical tests and models reported in the manuscript results and figure legends. All p values reported were obtained from two-sided test.

a. *On day 2 after surgery, partial IRI mice displayed significantly increased urinary albumin excretion (albumin-creatinine-ratio (ACR))*

   Mean ± SEM: 24357.5 ± 10073.2 vs 3333.1 ± 786.3 mg/g, n = 4 and 3 for partial IRI vs sham treatment. $\Delta = 21024$, 95% CI: [35099, 6949], $t\,(32) = 4.29$, $p = 0.0011$

b. *Urinary albumin to creatinine ratio and glomerular filtration rate (GFR) measured in sham and partial IRI mice* – fig. 1b and c

   Statistical test: repeated measurement 2-way ANOVA, factors: treatment and days from treatment, post-hoc analysis: multiple comparisons, Bonferroni correction (b: n = 3 and 4 mice and c: n = 4 and 8 mice for sham and partial IRI, respectively).

   (b) Main fixed effect: Time x Treatment, $p = 0.0201$, $F\,(6, 27) = 3.070$

   (c) Main fixed effect: Time x Treatment, $p = 0.2625$, $F\,(7, 65) = 1.304$

   Multiple comparisons unique significant difference: day 2 (b), $t\,(32) = 4.29$, $p = 0.0011$

c. *Quantification of PI+ nuclei in % of the total nuclei number per individual tubular segment – fig. 1g*

   Mean ± 95% CI with scatterplot (n = 123, 334, and 263, from 5, 7, and 6 mice for Not-IR, Mid, and IR, respectively).

   Statistical test: linear mixed-effect model, response: PI+ nuclei (%), fixed effect: partial IRI area ($F\,(2, 717) = 135.29$, $p < .001$), random effect: mouse ID.

d. *In Mid regions, significantly less injury was detectable than in IR regions*

Mean ± SEM: 6.21 ± 0.54% vs 21.78 ± 1.32% necrotic cells, n = 334 and 263 tubule segments from 7 and 6 mice from Mid and IR, respectively. Δ = -15.68, 95% CI: [-18.01, -13.34], t (717) = -13.18, p < 0.001

e. *In Not-IR regions, the extent of damage was not significantly different from zero*

Mean ± SEM: 0.15 ± 0.07% necrotic cells, n = 123 tubule segments from 5 mice.

Δ = 2.2, 95% CI: [6.5078, -1.9127], t (717) = 1.07, p = 0.284

f. *Assessment of nephron segment identity by quantitative fluorescence emission – fig. 2b*

Boxplot, line at median, edges at 25th and 75th percentiles, whiskers from min to max, individual points considered outliers (n = 152, 190 and 38 PT-S1, PT-S2 and DCT/CD segments, respectively, from 4 partial IRI mice, 24h post partial IRI.

Statistical test: linear mixed-effect model, response: fluorescent intensities, fixed effect: segment identity (F (2, 377) = 118.9, 26.47, 94.23 for λ_{Em} = 500-550 nm, λ_{Em} = 435-485 nm, and their ratio, respectively, p < .001 for all), random effect: mouse ID.

Multiple comparisons where p> 0.001: Blue emission, Ch4: PT-S2 vs. DCT/CD, t (377) = -2.857: p = 0.0045; Ratio PT-S1 vs. DCT/CD, t (377) = -3.056: p = 0.002).

g. *Quantification of PI+ nuclei along different nephron regions, in % of the total nuclei number per individual tubular segment in IR-region of partial IRI kidneys – fig. 2f and g*

Mean ± 95% CI with scatterplot (n = 195, 90, and 105 from 6 mice for PTs, PT-S1, and PT-S2, respectively, and 14 for DCT/CD from 4 mice).

Statistical test: linear mixed-effect model, response: PI+ nuclei (%), fixed effect: segment identity (F: $F_{(1,207)} = 20.30$, $p < .001$, G: $F_{(1,206)} = 24.46$, $p < .001$), random effect: mouse ID.

h. *Necrotic cell death in DCT/CDs of IR regions was overall of low extent compared to PT segments*

   Mean ± SEM: 2.89 ± 0.75% vs 20.41 ± 1.49% necrotic cells, n = 195 (PTs, from 6 mice) and 14 (DCT/CDs, from 4 mice) tubule segments. Δ = -22.56, 95% CI: [-32.43, -12.69], $t_{(207)} = -4.50$, $p < 0.001$

i. *We observed a significantly higher degree of necrotic cell death in PT-S1 segments than PT-S2 segments*

   Mean ± SEM: 26.19 ± 2.45% vs 15.45 ± 1.68% PI-positive nuclei, n = 90 and 105 tubule segments from 6 mice. Δ = 12.35, 95% CI: [7.56, 17.14], $t_{(206)} = -5.08$, $p < 0.001$

j. Quantification of GFP-expression (in % of total nuclei per segment) across different experimental groups – *fig. 3 and S5*

   Mean ± 95%.CI (fig.3) and boxplot, line at median, edges at 25th and 75th percentiles, whiskers from min to max, individual points considered outliers (fig. S5). Statistical test: linear mixed-effect model, response: GFP+ nuclei (%), fixed effects: days from partial IRI in all, interaction with treatment (b, $F_{(7, 5444)} = 45.566$, $p < .001$), area of partial IRI (c, $F_{(14, 2967)} = 10.053$, $p < .001$) and segment identity (d, $F_{(14, 2428)} = 4.996$, $p < .001$), random effect: mouse ID

k. Cumulative quantification of GFP-expressing nuclei (in % of total nuclei number per segment) which arose either in close proximity (< 25 μm) or in distant localization (>25 μm) relative of any propidium iodide (PI)+ cell detected on day 0 in the same tubule segment – *fig. 4c*

Mean ± 95%CI with scatterplot. (n = 145, 184 and 37 segments from 4 mice for PT-S1, PT-S2, and DCT/CD, respectively). Statistical test: paired t-test of proximal (<25 μm) vs distant (>25 μm) GFP+ nuclei, $p < .05$ considered as significant, test statistics: PT-S1: Δ = 2.43, 95% CI: [1.90, 2.95], $t(433) = 9.128$, $p < 0.001$, PT-S2: Δ = -0.5331, 95% CI: [-1.15, 0.08], $t(547) = -0.53$, $p = 0.0908$, DCT/CD: Δ =-0.5 , 95% CI: [-0.89, -0.13], $t(109) = -2.681$, $p = 0.0085$

l. *We observed a significantly higher count of epithelial GFP-cells located within immediate proximity of PI-positive nuclei (2.27 ± 0.15%) as compared to those detected in distant localization (1.62 ± 0.14%).*

Mean ± SEM: 2.27 ± 0.15% vs 1.62 ± 0.14% GFP-positive nuclei (%), n = 336 segments from 4 mice. Paired t-test, Δ = 0.65, 95% CI: [0.26, 1.03], $t\,(1091) = 3.30$, $p < 0.001$.

m. *Dynamic quantification of GFP-expressing nuclei (in % of total nuclei number per segment) for PT-S1, PT-S2, and DCT/CD, which arose at days 1, 2, and 3 after partial IRI either in close proximity (< 25 μm) or in distant localization (>25 μm) relative of any propidium iodide (PI)+ cell detected on day 0 in the same tubule segment– fig. 4d*

Mean ± 95% CI with scatterplot. Detailed information on sample size: Supplementary Table 3. Statistical test: linear mixed-effect model, response: proximal and distant GFP+ nuclei (%), fixed effect: days from partial IRI, interaction with segment identity (< 25 μm, $F\,(4, 1083) = 1.77$, $p < .001$; >25 μm, $F\,(7, 1083) = 9.02$, $p < .001$), random effect: mouse ID.

n. *Quantification of tubular GFP-expression in PT-S1 and PT-S2 segments clustered by damage threshold– fig. 4e*

Mean ± 95% CI with scatterplot. n = 223, 140, 157 PT-S1 and 250, 237, 103 PT-S2 for non necrotic, moderare, and severe, respectively, from 6 mice. Statistical test: linear mixed-effect model, response: GFP+ nuclei (%), fixed effects: segment identity, days from partial IRI, and injury group. Effect of interactions: (F (6, 1086) = 2.46, p = 0.022). Random effect: mouse ID.

o. Luminal granular cast area in PT-S1 and PT-S2 nephron segments– *fig. 5c and d*

(c). Mean ± 95% CI with scatterplots. N= 468, 328, 329, 320 for day 0, 1, 2 and 3 from 6 (day 00) and 4 (day 01-03) mice, respectively. Statistical test: linear mixed-effect model, response: granular cast area (%), fixed effect: day from partial IRI (F (3, 1441) = 18.946, p < .001). Random effect: mouse ID.

(d). Mean ± 95% CI with scatterplots. Statistical test: linear mixed-effect model, response: granular cast area (%), fixed effect: segment type (F (1, 973) = 50.77, p < .001). Random effect: mouse ID.

p. Dynamic assessment of albumin reuptake ability of sham, uninjured, recovered, and atrophic S1 proximal tubule (PT-S1) segments over time – *fig.* 7b

Mean ± 95% CI with scatterplot. n = 80, 40, 28, and 22 segments from sham, uninjured, recovered, and atrophic tubules, respectively from n = 3 sham and n = 4 partial IRI mice. Statistical test: linear mixed-effect model, response: alexa594-albumin ratio in tubular cytoplasm/plasma, fixed effects: days from partial-IRI, interaction with tubules´ fate (F (9, 583) = 9.563, p < .001), random effect: mouse ID.

q. *Quantification of PI+ nuclei and GFP+ nuclei (% of total nuclei per segment) in relation to tubular fate - fig.* 7c-*d*

Mean ± 95% CI with scatterplot. (c): n = 89 and 92 recovered and atrophic tubules from 4 and 3 mice, respectively. (d): n = 124 and 168 from 4 mice. Statistical tests: linear mixed-effect model, response: PI+ (c) and GFP+ (d) nuclei (%), fixed effect: fate (F (1, 179) = 31.454, p<.001 in c, F (1, 290)= 7.5114, p=0.006 in d), random effect: mouse ID.

r. *Atrophic tubules demonstrated significantly more necrosis on day 0 as compared to recovering tubules.*

Mean ± SEM: 23.78±1.54% vs 13.29±1.04% PI-positive nuclei (%), n = 92 and 89 from 3 and 4 mice for atrophic and recovering tubules. Δ = 10.48, 95% CI: [6.79, 14.17], $t(179) = 5.60$, $p < 0.001$.

s. *We found significantly higher GFP-expression in tubules that turned atrophic as compared to recovering tubules*

Mean ± SEM: 10.85 ± 0.94% vs. 4.92 ± 0.54% GFP-positive nuclei, n = 168 and 124 segments from 4 mice. Δ = 3.71, 95% CI: [1.04, 6.37], t (290) = 2.74, p = 0.0065.

t. *Necrotic injury distribution along the nephron in IR and Mid areas- fig. S1 b and c*

b: Quantification of PI+ nuclei in % of the total nuclei number per individual tubular segment. Boxplot, line at median, edges at 25th and 75th percentiles, whiskers from min to max, individual points considered outliers (n = 484, 42, 226, 258 segments from 7 mice for PT, DCT/CD, PT-S1, and PT-S2, respectively). Statistical test: linear mixed-effect model,

response: PI+ nuclei (% of total nuclei/segment), fixed effect: segment identity (top: F (1, 524) = 16.72, $p < .001$, bottom: F (2, 523) = 20.22, $p < .001$), random effect: mouse ID.

c: Quantification of PI+ nuclei determined from a 2D plane at the highest cross-sectional area within each tubular segment, normalized by respective cross-sectional area. Statistical test: linear mixed-effect model, response: cross-sectional PI+ nuclei over maximal cross-sectional area (%), fixed effect: segment identity (F (2, 368) = 12.309, $p < .001$), random effect: mouse ID.

u. Quantification of the average GFP-expression following a selective PT-S1 injury - *fig. 6*

d: Boxplot, line at median, edges at 25th and 75th percentiles, whiskers from min to max, individual points considered outliers. n = 461 and 39 for uncasted and casted segments, respectively, from 7 mice. Statistical test: linear mixed-effect model, response: GFP+ nuclei (%), fixed effect: casting state (F (1, 498) = 273.8, $p < .001$), random effect: mouse ID.

f: Boxplot, line at median, edges at 25th and 75th percentiles, whiskers from min to max, individual points considered outliers. n = 20 for each zone from 7 mice. Statistical test: linear mixed-effect model, response: GFP+ nuclei (n. of nuclei / μm^2), fixed effect: distance from laser injury (μm) group, random effect: mouse ID. Statistical comparison to site of laser injury area (0-35 μm): 35-85 μm, 95%CI: [-0.0001716, 0.00019633], $t(76) = 0.14$, p = 0.89; 85-135 μm, 95%CI: [-0.00032812, 3.981e-05], t(76) = -1.56, p= 0.12; > 135 μm, 13595%CI: [-0.00043531, -6.7382e-05], t(76) = -2.72, p= 0.008

Days from procedure	Day 0				Day 1				Day 2				Day 3				Day 4				Day 7				Day 14				Day 21			
**Experimental group**	mean	±	SEM	n	mean	±	SEM	n	mean	±	SEM	n	mean	±	SEM	n	mean	±	SEM	n	mean	±	SEM	n	mean	±	SEM	n	mean	±	SEM	n
Partial-IR<br>9 mice	0.26	±	0.05	*543*	1.29	±	0.18	*380*	4.44	±	0.36	*380*	5.18	±	0.35	*520*	2.02	±	0.27	*264*	1.21	±	0.19	*350*	0.55	±	0.12	*287*	0.29	±	0.06	*267*
Sham<br>3 mice	0.37	±	0.06	*322*	0.06	±	0.03	*322*	0.22	±	0.05	*322*	0.19	±	0.04	*322*	0.06	±	0.03	*322*	0.11	±	0.05	*322*	0.62	±	0.08	*322*	0.58	±	0.1	*215*
**Partial IRI regions**																																
IR<br>13 FOVs	0.15	±	0.05	*181*	3.3	±	0.48	*119*	7.88	±	0.67	*119*	7.06	±	0.64	*172*	4.37	±	0.9	*55*	1.69	±	0.44	*104*	0.75	±	0.33	*88*	0.14	±	0.08	*80*
Mid<br>16 FOVs	0.37	±	0.08	*274*	0.51	±	0.11	*187*	3.58	±	0.53	*187*	5.39	±	0.53	*253*	1.88	±	0.31	*151*	1.29	±	0.26	*188*	0.5	±	0.13	*156*	0.16	±	0.05	*138*
Not-IR<br>7 FOVs	0.15	±	0.06	*88*	0.02	±	0.02	*74*	1.06	±	0.3	*74*	1.22	±	0.28	*95*	0.14	±	0.08	*58*	0.08	±	0.05	*58*	0.27	±	0.13	*43*	0.9	±	0.27	*49*

Supplementary Table 1: Dynamic volumetric quantification of GFP+ nuclei (% of total nuclei/ segment) as determined per experimental group (sham and partial IRI) and per partial IRI region (Not-IR, Mid, IR) over 3 weeks. Data expressed as mean ± SEM. n: number of tubule segments included in each group.

**Days from partial IRI**	PT-S1				PT-S2				DCT/CD			
	mean	±	SEM	n	mean	±	SEM	n	mean	±	SEM	n
**Day 0** (8 mice)	0.41	±	0.10	*220*	0.16	±	0.05	*272*	0.15	±	0.09	*51*
**Day 1** (4 mice)	1.93	±	0.38	*152*	1.02	±	0.17	*190*	0.05	±	0.05	*38*
**Day 2** (4 mice)	3.91	±	0.58	*152*	5.57	±	0.55	*190*	0.92	±	0.27	*38*
**Day 3** (6 mice)	4.02	±	0.43	*212*	6.68	±	0.58	*260*	2.19	±	0.62	*48*
**Day 4** (4 mice)	1.61	±	0.29	*114*	2.64	±	0.50	*125*	0.79	±	0.35	*25*
**Day 7** (5 mice)	0.84	±	0.24	*154*	1.58	±	0.33	*166*	1.03	±	0.51	*30*
**Day 14** (4 mice)	0.45	±	0.14	*128*	0.69	±	0.23	*135*	0.25	±	0.25	*24*
**Day 21** (5 mice)	0.28		0.09	*122*	0.25		0.08	*124*	0.60		0.41	*21*

Supplementary Table 2: Distribution of GFP+ nuclei (in % of total nuclei/ segment) along the nephron. Data expressed as mean ± SEM. n: number of partial-IRI subjects imaged at indicated days. n: number of tubules included in each group.

Days from partial IRI	Segment	GFP+ nuclei (%) < 25 µm of necrotic sites				GFP+ nuclei (%) > 25 µm of necrotic sites			
		mean	±	SEM	n	mean	±	SEM	n
Day 1	PT-S1	1.95	±	0.38	*145*	0.06	±	0.05	*145*
	PT-S2	0.77	±	0.15	*184*	0.28	±	0.06	*184*
	DCT/CD	0.00	±	0.00	*37*	0.05	±	0.05	*37*
Day 2	PT-S1	3.74	±	0.56	*145*	0.34	±	0.09	*145*
	PT-S2	2.69	±	0.35	*184*	3.06	±	0.42	*184*
	DCT/CD	0.08	±	0.06	*37*	0.86	±	0.28	*37*
Day 3	PT-S1	2.95	±	0.42	*144*	0.95	±	0.15	*144*
	PT-S2	3.17	±	0.42	*180*	4.91	±	0.62	*180*
	DCT/CD	0.42	±	0.24	*36*	1.15	±	0.42	*36*

Supplementary Table 3: Spatial distribution of GFP+ nuclei (% of total nuclei/ segment, assessed in 3D) relative to necrotic sites. Data expressed as mean ± SEM. n: number of tubule segments included in each group. Data for each day was obtained from 4 subjects.

Treatment	Partial-IRI			Sham		
**Protocol**	IVM - n = 11(M)		GFR - n = 4(M) 4(F)	IVM - n = 3 (M)		GFR - n = 3(M) 1(F)
	Unique Mice (FOV)	Unique Segments	Unique Mice	Unique Mice (FOV)	Unique Segments	Unique Mice
Day 0	8 (35)	757	8	3 (11)	400	4
Day 1	4 (20)	389	8	3 (11)	400	4
Day 2	4 (20)	389	-	3 (11)	400	-
Day 3	8 (35)	743	-	3 (11)	400	-
Day 4	4 (16)	326	8	3 (11)	400	4
Day 7	5 (20)	417	8	3 (11)	400	4
Day 14	4 (16)	354	8	3 (11)	400	4
Day 21	5 (15)	336	8	2 (8)	293	4
Day 28	-	-	8	-	-	4
Day 49	-	-	4	-	-	3

Supplementary Table 4: Study design. M: male. F: female.
