# Supplementary material for: Longitudinal tracking of acute kidney injury reveals injury propagation along the nephron
Source: Nat Commun. 2023 Jul 21;14:4407. doi: 10.1038/s41467-023-40037-y (PMC10362041; doi:10.1038/s41467-023-40037-y)
Supplement: Supplementary file 3 — Description of Additional Supplementary Files [file 41467_2023_40037_MOESM3_ESM.pdf]

Description of Additional Supplementary Files

Supplementary Movie 1: Injury propagation along the nephron. Registered serial in vivo 2-photon microscopy images of a representative Mid region in a CycB1-GFP reporter kidney at day 0, 3, 7 and 14 after partial IRI. Clustered tubule necrotic cell death (⊥) is followed by tubule proliferation (arrowheads), which first emerges at the site of injury and then expands towards initially non-necrotic epithelium, which finally results in tubule atrophy on day 7. Arrows: granular casts. ω: atrophic tubules. Scale bar: 50 μm

Supplementary Movie 2: Representative Mid region. Registered serial in vivo 2-photon microscopy images of a representative Mid region in a CycB1-GFP reporter kidney at day 0, 3, 7, 14 and 21 after partial IRI. ⊥: necrotic nuclei. Arrowheads: GFP-expression. Scale bar: 50 μm.

Supplementary Movie 3: Representative IR region. Registered serial in vivo 2-photon microscopy images of a representative IR region in a CycB1-GFP reporter kidney at day 0, 3, 7 and 14 after partial IRI. ⊥: necrotic nuclei. Arrowheads: GFP-expression. Arrows: granular casts. ω: atrophic tubules. Scale bar: 50 μm.
