# Supplementary material for: Longitudinal tracking of acute kidney injury reveals injury propagation along the nephron
Source: Nat Commun. 2023 Jul 21;14:4407. doi: 10.1038/s41467-023-40037-y (PMC10362041; doi:10.1038/s41467-023-40037-y)
Supplement: Supplementary file 7 — Reporting Summary [file 41467_2023_40037_MOESM7_ESM.pdf]

nature portfolio

Corresponding author(s): Ina Maria Schiessl

Last updated by author(s): Jun 20, 2023

Reporting Summary

Nature Portfolio wishes to improve the reproducibility of the work that we publish. This form provides structure for consistency and transparency in reporting. For further information on Nature Portfolio policies, see our Editorial Policies and the Editorial Policy Checklist.

Statistics

For all statistical analyses, confirm that the following items are present in the figure legend, table legend, main text, or Methods section.

n/a	Confirmed	
☐	☒	The exact sample size (*n*) for each experimental group/condition, given as a discrete number and unit of measurement
☐	☒	A statement on whether measurements were taken from distinct samples or whether the same sample was measured repeatedly
☐	☒	The statistical test(s) used AND whether they are one- or two-sided<br>*Only common tests should be described solely by name; describe more complex techniques in the Methods section.*
☐	☒	A description of all covariates tested
☐	☒	A description of any assumptions or corrections, such as tests of normality and adjustment for multiple comparisons
☐	☒	A full description of the statistical parameters including central tendency (e.g. means) or other basic estimates (e.g. regression coefficient) AND variation (e.g. standard deviation) or associated estimates of uncertainty (e.g. confidence intervals)
☐	☒	For null hypothesis testing, the test statistic (e.g. *F*, *t*, *r*) with confidence intervals, effect sizes, degrees of freedom and *P* value noted<br>*Give P values as exact values whenever suitable.*
☒	☐	For Bayesian analysis, information on the choice of priors and Markov chain Monte Carlo settings
☒	☐	For hierarchical and complex designs, identification of the appropriate level for tests and full reporting of outcomes
☐	☒	Estimates of effect sizes (e.g. Cohen's *d*, Pearson's *r*), indicating how they were calculated

Our web collection on statistics for biologists contains articles on many of the points above.

Software and code

Policy information about availability of computer code

Data collection	PraireView IV software<br>Fluoview FV31S
Data analysis	Microsoft Excel, version 2305 Build 16.0.16501.20074<br>ImageJ 1.53f51<br>MATLAB_R2022a<br>GraphPad Prism, version 9.5.1 (733)<br><br>All analysis codes used for statistical analyses and generation of plots in MATLAB are available in the Zenodo repository: https://zenodo.org/record/7892132#.ZFNUNKBBwuU

For manuscripts utilizing custom algorithms or software that are central to the research but not yet described in published literature, software must be made available to editors and reviewers. We strongly encourage code deposition in a community repository (e.g. GitHub). See the Nature Portfolio guidelines for submitting code & software for further information.

Data

Policy information about availability of data

All manuscripts must include a data availability statement. This statement should provide the following information, where applicable:

- Accession codes, unique identifiers, or web links for publicly available datasets
- A description of any restrictions on data availability
- For clinical datasets or third party data, please ensure that the statement adheres to our policy

Source data are provided with this paper. The two-photon imaging data generated in this study have been deposited in the DataDryad database (doi:10.5061/dryad.vq83bk3z8): https://datadryad.org/stash/share/NuiNLT6qTdKR74FSQkXL2PAlnwyOFT_5T7Cy8t8NzeU

Human research participants

Policy information about studies involving human research participants and Sex and Gender in Research.

Reporting on sex and gender	N/A
Population characteristics	N/A
Recruitment	N/A
Ethics oversight	N/A

Note that full information on the approval of the study protocol must also be provided in the manuscript.

Field-specific reporting

Please select the one below that is the best fit for your research. If you are not sure, read the appropriate sections before making your selection.

- [x] Life sciences
- [ ] Behavioural & social sciences
- [ ] Ecological, evolutionary & environmental sciences

For a reference copy of the document with all sections, see nature.com/documents/nr-reporting-summary-flat.pdf

Life sciences study design

All studies must disclose on these points even when the disclosure is negative.

Sample size

The sample size for each experimental group was determined by: 1.) Previous experience of performing experiments involving implantation of abdominal imaging window (AIW), 2.) trial experiments of partial IRI models, and 3.) trial experiments of sham-operated mice.
Power calculations were not performed before experimental collection in this study since the parameters we quantified (degree of necrosis, degree of proliferation, albumin reuptake over time, etc...) resulted from a completely novel experimental approach where the same subject has been imaged longitudinally for up to 3 weeks after AIW implantation- and, therefore, we could not estimate variances of the measurements with sufficient confidence before accumulating preliminary data. However, sample sizes were sufficient to detect significant changes, and a minimum of 3 subjects was allocated in each experimental group.

Serial intravital microscopy of partial IRI animals was challenged by strong tissue remodeling that sometimes caused missing data points over time. To compensate for missing data points, we included higher n-numbers in the partial IRI group. As outlined in the methods, we included all serially imaged data collected from any subject into data analysis, whenever successfully generated.

Data exclusions

Partial IRI mice were included in the study, upon visual confirmation of temporal restriction and subsequent re-installment of blood flow in approximately half of the kidney tissue (as shown in fig. 1D). Partial IRI mice were excluded from the study, if incorrect placement of the abdominal imaging window did not allow identification of MID or IR regions using intravital microscopy.

In cases of unsuccessful re-identification of individual FOVs during any acquisition time point, serial data from respective time points was missing and could not be included in further analysis. Displayed data state n numbers for analyzed tubule segments and experimental mice respectively.

Replication

Statistics and Reproducibility
Intravital imaging experiments of individual experimental mice were performed as independent experiments. Upon reassurance that inclusion criteria were given (successful partial IRI surgery and correct placement of the abdominal imaging window implant), different partial IRI regions (Not-IR, Mid, and IR) were reproducibly identifiable based on distinct necrotic cell damage patterns on day 00. The biological phenomena described in this study were reproducible in all attempted experiments.
Analysis of albumin-creatinine ratio from urine samples were performed as duplicates. Baseline transcutaneous GFR measurements were

performed as duplicates. After IRI/sham surgery, transcutaneous GFR measures were performed longitudinally and were hence obtained as single values per time point and animal.

Representative serial in vivo 2-photon images were selected from data obtained from the following number of independent experiments: partial IRI: day 0 = 8 mice, day 1 - 2 = 4 mice, day 3 = 8 mice, day 4 = 4 mice, day 7, 14, 21 = 4 mice; and sham: day 0, 1, 2, 3, 4, 7, 14 = 3 mice, day 21 = 2 mice. Representative correlative in vivo and ex vivo images for classification of remodeling processes were selected from data obtained from 3 independent experiments. Representative correlative in vivo and ex vivo images for detection of VCAM1-positivity in atrophic tubules were selected from data obtained from 3 independent experiments.

Randomization

Subjects were randomly assigned to undergo either partial IRI or sham surgery. Further assignment to GFR or intravital microscopy experiments (including sub-assignment to short-term or long-term imaging groups) was also done randomly.

Blinding

Blinding of experimental treatment could not be performed in our study design as the validation of the expected surgical outcome required close microscopic examination during surgery (as explained above in "Data Exclusion" and "Replication").

Reporting for specific materials, systems and methods

We require information from authors about some types of materials, experimental systems and methods used in many studies. Here, indicate whether each material, system or method listed is relevant to your study. If you are not sure if a list item applies to your research, read the appropriate section before selecting a response.

Materials & experimental systems

n/a	Involved in the study	
☐	☒	Antibodies
☒	☐	Eukaryotic cell lines
☒	☐	Palaeontology and archaeology
☐	☒	Animals and other organisms
☒	☐	Clinical data
☒	☐	Dual use research of concern

Methods

n/a	Involved in the study	
☒	☐	ChIP-seq
☒	☐	Flow cytometry
☒	☐	MRI-based neuroimaging

Antibodies

Antibodies used

Rabbit anti-VCAM-1 (Abcam AB0134047, 1:200) primary antibody;
Donkey anti-Rabbit Alexa 405 (Jackson ImmunoResearch 711-175-152, 1:500) secondary antibody

Validation

Primary antibody Rabbit anti-VCAM-1 was diluted as previously published [1, 2].
Primary antibody Rabbit anti-VCAM-1 specificity for immunohistology in mouse tissue was validated by the manufacturer using knockout validation and mouse spleen tissue as positive control. Suitability of Rabbit anti-VCAM-1 for identifying cells of failed tubule repair was previously established for mouse and human tissue [1, 2].
[1] Kirita, Y., et al., Cell profiling of mouse acute kidney injury reveals conserved cellular responses to injury. Proc Natl Acad Sci U S A, 2020. 117(27): p. 15874-15883.
[2] Muto, Y., et al., Single cell transcriptional and chromatin accessibility profiling redefine cellular heterogeneity in the adult human kidney. Nat Commun, 2021. 12(1): p. 2190.

Animals and other research organisms

Policy information about studies involving animals; ARRIVE guidelines recommended for reporting animal research, and Sex and Gender in Research

Laboratory animals

Tg(Pgk1-Ccnb1/EGFP)1Aklo (CycB1-GFP) reporter mice were purchased from The Jackson Laboratory (Strain #:023345) and bred in the barrier animal facilities at the Department of Biomedicine, Aarhus University.

Animals used for partial IRI in vivo experiments were 17.7 ± 2.3 weeks of age (mean ± SEM)
Animals used for selective laser-induced PT-S1 injury were 8.7 ± 1.1 weeks (mean ± SEM)

For confirmation of reliable PT-S1 and PT-S2 classification four 10-week-old C57/Bl6 mice were used

Wild animals

Not used for the present study.

Reporting on sex

It is widely known that females respond to ischemia-reperfusion injury with a milder phenotype. We therefore collected data mainly from male animals.
n= 21 male subjects
n= 5 female subjects.
We did not detect differences between the data obtained from male and female subjects, but as sex comparison was not considered

	in the original study design, the n-numbers don't allow final conclusions.<br><br>For laser-induced injury of specifically PT-S1 segments, we used 7 male mice.<br><br>For confirmation of reliable PT-S1 and PT-S2 classification four male mice were used.
Field-collected samples	Not used for the present study.
Ethics oversight	All experimental procedures involving animals in this study were approved by local authorities (Animal Experimental Inspectorate, Denmark, permit number: 2020-15-0201-00443)

Note that full information on the approval of the study protocol must also be provided in the manuscript.
