# Supplementary material for: Longitudinal tracking of acute kidney injury reveals injury propagation along the nephron
Source: Nat Commun. 2023 Jul 21;14:4407. doi: 10.1038/s41467-023-40037-y (PMC10362041; doi:10.1038/s41467-023-40037-y)
Supplement: Supplementary file 8 — Source Data [file 41467_2023_40037_MOESM8_ESM.zip › SourceData_ReadMe.docx]

Description of SourceData File provided for the manuscript *Longitudinal tracking of acute kidney injury reveals injury propagation along the nephron.*

The *SourceData.xlsx* file contains individual excel sheets named after each manuscript figures and/or subplot. The numbers shown in the *SourceData.xlsx* file have been extracted from imaging data as described in the *Materials and Methods* section of the manuscript. The original imaging dataset is freely available for download on Dryad (<https://doi.org/10.5061/dryad.vq83bk3z8>).

The data provided in *SourceData.xlsx* file have been analyzed on MATLAB v. 2022a (© The Mathworks Inc.) and GraphPad Prism v.9.5.0 (© 1994 - 2022 GraphPad Software, LLC.). The authors recommend any user interested in processing the data provided in the manuscript to use the MATLAB scripts provided with the *SourceData.xlsx* table. The set of MATLAB scripts we used to analyze the data is freely available for download on Zenodo (<https://zenodo.org/record/7892132>).

Similarly, to the table itself each .m code file is named using specific indication of the figure and/or subplot generated upon running the script. Each script is provided with comments and documentations which, we believe, will allow an entry-level MATLAB user to understand, modify, and perform at need each numerical analysis, statistical test and plot showed in the manuscript. In order to start processing the *SourceData.xlsx* numerical results on MATLAB, users are required to open the “format_table.m” file on MATLAB and import the *SourceData.xlsx* table as indicated by the instructions in line 1-23.

Essential Terminology – cells having light blue color denote essential identifiers and numerical information used for successive normalization of other measured data:

- *Mouse ID*: individual subject identifier
- *Follow*-Up*:* intravital imaging group (3 days or 3 weeks imaging)
- *Surgery*: IRI (for *partial IRI surgical experiments*), CTR (for *Sham controls*)
- *Area*: Mid, IR and Not-IR as defined by the partial IRI disease model
- *FOV*: categorical identifier of a given field-of-view (FOV) for each Mouse-ID
- *Segment*: categorical identifier of a given tubular epithelial segment for each combination of Mouse-ID and unique FOV.
  - The unique statistical unit for results of the imaging experiments was a tubular epithelial segment. Therefore, each row of the *SourceData* table corresponds of a unique combination of a given *Mouse-ID, FOV,* and *Segment*
- *Type*: segments identity within the nephron, classified as described in Fig. 2A-C of the manuscript*. PT-S1 and PT-S2*: proximal tubule segment S1 and S2. sCDT: collecting ducts and distal convoluted ducts (DCT/CD). *UNC:* unclassified
- *PI+/Total*: % of PI+ nuclei over total nuclei for a given tubule. The number of *measured* PI nuclei is reported in the column *PI+ nuclei (tubulus) – Day 0 (number*).
- *Signal Chx*: absolute signal intensity of a given channel using a 750 nm excitation acquisition protocol.
- *Area of Segment (µm^2) -Calc.Day0. Depth: 15 µm fr. Top*: measured crossectional area of any tubular segment.

Other terminology specifically refers to specific results and analyses reported in individual figures in the manuscript and are documented on the MATLAB figure-specific scripts provided with *SourceData*.
